# Evaluating the Prognostic Significance of the H-Index and Systemic Inflammatory Parameters in IDH-Wildtype Glioblastoma Survival

**DOI:** 10.3390/curroncol33100604

**Published:** 2026-10-08

**Authors:** Havva Yeşil, Bekir Demirel, Ercan Gumusburun

**Affiliations:** 1 Department of Medical Oncology, Faculty of Medicine, Gaziantep University, 27310 Gaziantep, Türkiye; 2 Department of Internal Medicine, Faculty of Medicine, Gaziantep University, 27310 Gaziantep, Türkiye

**Keywords:** IDH-wildtype glioblastoma, H-index, HALP score, AISI, systemic inflammation, prognosis

## Abstract

**Background:** IDH-wildtype Glioblastoma is characterized by an aggressive clinical course. This study aimed to evaluate the prognostic value of the H-index, the hemoglobin-albumin-lymphocyte-platelet (HALP) score and the aggregate index of systemic inflammation (AISI). **Methods:** We retrospectively analyzed 136 patients who underwent frontline chemoradiotherapy. **Results:** Optimal cut-offs were 20.94 for HALP, 2.29 for H-index, and 989.64 for AISI. A high H-index was associated with shorter overall survival (OS) in univariate analysis (*p* = 0.007) and showed the highest time-dependent AUC for OS among the three indices. In multivariable Cox models, comorbidities (*p* = 0.043), subtotal resection (*p* < 0.001), corticosteroid use (*p* = 0.040), and a low HALP score (*p* = 0.012) were associated with poor OS, whereas the associations for a high H-index (*p* = 0.084) and a high AISI (*p* = 0.036) did not meet the Bonferroni-adjusted significance threshold. In a sensitivity analysis restricted to patients not receiving corticosteroids (*n* = 80), H-index-based stratification remained associated with OS (median 14.4 vs. 24.9 months, log-rank *p* = 0.025), whereas the association for AISI was not retained. **Conclusions:** The H-index, evaluated here for the first time, and the HALP score may provide complementary prognostic information. Given the retrospective single-center design, the potential influence of corticosteroids, and the data-driven cut-offs, these findings should be regarded as exploratory and require external validation before clinical application.

## Simple Summary

IDH-wildtype Glioblastoma is a highly aggressive brain cancer that grows rapidly and changes the body’s normal defense systems. Clinicians currently find it difficult to accurately predict how long a patient will survive using standard tests alone. This study investigated whether common components found in routine blood tests, which reflect a patient’s nutrition and internal inflammation levels, could help predict survival outcomes. By reviewing medical records, we found that some combinations of these blood cells and proteins were associated with shorter survival. Because these indicators are inexpensive and routinely available, they may help clinicians better understand the course of the disease. However, some of these indicators can be influenced by corticosteroid treatment, and our findings need to be confirmed in other patient groups before they can be used in routine care.

## 1. Introduction

Isocitrate dehydrogenase (IDH)-wildtype glioblastoma, defined as a diffuse astrocytic tumor, represents the most prevalent primary malignant neoplasm of the central nervous system in adult populations [1,2]. Without targeted therapeutic interventions, the life expectancy of individuals diagnosed with this aggressive disease is severely constrained to a few months. Furthermore, our understanding aligns with the landmark study by Stupp et al., where the integration of external beam radiotherapy with concurrent and maintenance temozolomide (TMZ) chemotherapy has successfully extended the median overall survival threshold to approximately 15 months [3]. Despite the clinical implementation of these intensive multimodal therapeutic regimens, inevitable recurrence across patient cohorts restricts the five-year post-diagnostic survival rate to less than 5%, while 10-year long-term survivors remain exceedingly rare [2]. This profound treatment resistance highlights a critical high-mortality profile and underlines an urgent imperative for innovating novel therapeutic strategies.

For the vast majority of patients, the precise molecular and biological etiology of IDH-wildtype Glioblastoma remains largely elusive, which severely hinders the feasibility of early clinical detection [4]. While notable clinical advancements have been achieved in managing various alternative solid malignancies, frontline therapeutic options for IDH-wildtype Glioblastoma continue to exhibit marginal efficacy. This profound treatment resistance is primarily driven by the intricate tumor biology detailed by Virtuoso et al., charac-terized by an immunologically “cold”, non-inflamed microenvironment coupled with dense, reciprocal cellular interactions among neoplastic, vascular, stromal, and host immune compartments [5].

Persistent inflammatory cascades serve as critical drivers of oncogenesis and malignant progression, facilitating immune evasion, pathological angiogenesis, and therapeutic resistance, although their single diagnostic utility remains insufficient to fully decipher inter-individual heterogeneity [6,7]. Accumulating clinical evidence demonstrates that patient outcomes are dictated not only by autonomous tumor genetics but also by host-specific systemic biological responses, shifting focus toward multi-component biomarkers that encapsulate both dimensions [8]. Given their role in systemic inflammation, data from Hanggi and Ruffell show that distinct peripheral lineages—namely neutrophils, lymphocytes, monocytes, and platelets—exhibit specialized immunological traits within the circulation [9]. Parallel to these cellular dynamics, serum albumin accounts for a substantial proportion of protein reserves; functioning as a negative acute-phase biomolecule, its fluctuations accurately mirror host nutritional status and inflammatory stress, providing valuable predictive value regarding clinical outcomes [10,11].

Concurrently, accessible indices derived from routine peripheral blood profiles and biochemical assays offer vital prognostic and predictive insights for optimizing the long-term clinical monitoring of IDH-wildtype Glioblastoma patients. To date, various peripheral inflammation-based scores—including the neutrophil-to-lymphocyte ratio (NLR) validated by Guo et al. [12], platelet-to-lymphocyte ratio (PLR) by Bispo et al. [13], systemic immune-inflammation index (SII) by Yang et al. and Zhang and Ni [14,15], systemic inflammation response index (SIRI) by Topkan et al. [16], pan-immune-inflammation value (PIV) by Topkan et al. [17], Global Immune-Nutrition-Inflammation Index (GINI) by Aydin and Yuceer [18], hemoglobin-albumin-lymphocyte-platelet (HALP) score by Demir et al. [19], CRP–albumin–lymphocyte (CALLY) by Balci Topuz and Ozturk Iyilikci [20], and HALLMOUNT by Unlu et al. [21]—have been robustly established as independent predictors of progression-free survival (PFS) and overall survival (OS) in high-grade gliomas. Although the H-index has recently emerged as a highly promising prognostic biomarker in oral cavity cancer cohorts from Valero et al. [22], breast cancer from Seyyar et al. [23], and gallbladder carcinoma from Kattepur et al. [24]., its specific predictive utility and temporal kinetics have not yet been evaluated in the context of IDH-wildtype Glioblastoma.

The aim of this retrospective study was to thoroughly evaluate the prognostic significance of baseline H-index, HALP, and Aggregate Index of Systemic Inflammation (AISI) values in patients with IDH-wildtype Glioblastoma, with the ultimate goal of enhancing traditional risk stratification and optimizing personalized therapeutic strategies.

## 2. Materials and Methods

### 2.1. Patient Cohort and Institutional Review

This retrospective, single-center study evaluated a clinical cohort of 136 patients who underwent therapeutic intervention and subsequent follow-up at the Department of Medical Oncology, Gaziantep University Faculty of Medicine, between January 2014 and June 2025. Comprehensive demographic, clinical, laboratory, radiological, and histopathological datasets, along with long-term survival outcomes, were systematically extracted from institutional electronic health records and physical archival files. The analyzed variables encompassed gender, age, smoking status, comorbidities, extent of surgical resection (EOR), tumor location, tumor lateralization, multifocality, tumor diameter, Ki-67 index, corticosteroid use, anticonvulsant therapy, recurrence status, surgical intervention at recurrence, survival durations, baseline albumin levels, and hematological parameters—including absolute neutrophil, lymphocyte, monocyte, hemoglobin, and platelet counts.

The study protocol received formal approval from the Institutional Review Board of the Gaziantep University Ethics Committee (Approval No: 2026/289, Date: 10 June 2026) and strictly adhered to the ethical principles outlined in the Declaration of Helsinki (1964, amended in 2024).

### 2.2. Inclusion and Exclusion Criteria

Histopathological confirmation of adult-type diffuse glioma, specifically IDH-wildtype Glioblastoma, served as the primary diagnostic benchmark in accordance with the 5th edition of the World Health Organization (WHO) Classification of Tumors of the Central Nervous System. Eligible candidates met the following predefined inclusion criteria:

- Age and Pathology: Age ≥ 18 years at initial diagnosis with histologically verified IDH-wildtype Glioblastoma.
- Surgical Intervention: Attempted maximally safe surgical resection where anatomically and clinically feasible.
- Frontline Therapy: Completion of standard frontline concurrent chemoradiotherapy.
- Performance Status: Baseline Karnofsky Performance Status (KPS) score ≥ 70.
- Neuroimaging Availability: Availability of paired pre- and post-treatment gadolinium-enhanced magnetic resonance imaging (MRI) sequences.
- Laboratory Profile: Completion of baseline complete blood counts (CBC) and routine biochemical profiles.
- Comorbidities: Absence of concurrent active infections or a history of systemic immunosuppressive regimens.
- Secondary Malignancies: No prior or secondary malignancies, and no underlying end-organ failure.

**Figure 1:**
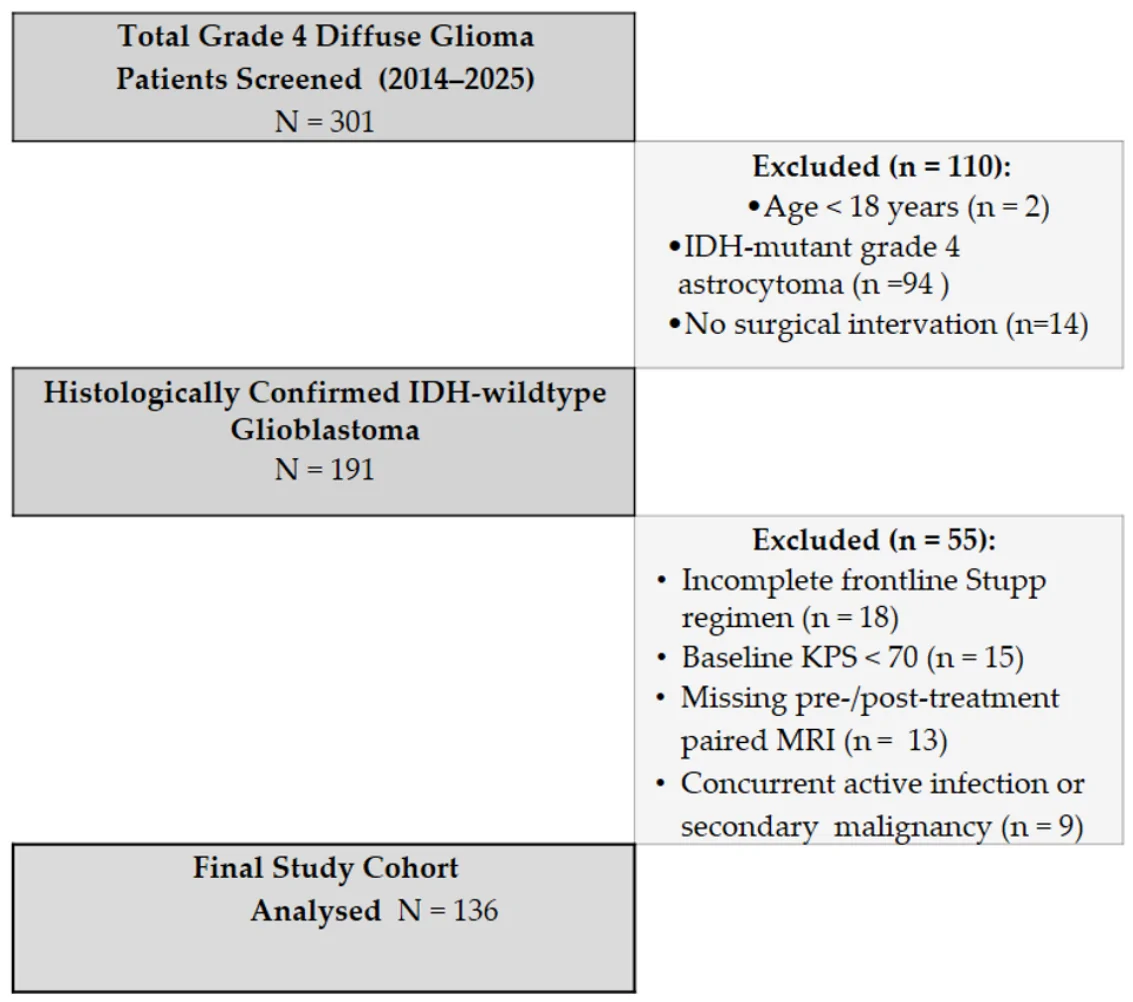
Patient Selection Flowchart.

The patient selection process is illustrated in Figure 1.

### 2.3. Therapeutic Protocol and Radiological Assessment

Standard-of-care management followed the established Stupp regimen [3]. Postoperative external beam radiotherapy was delivered at a total dose of 60 Gy in 30 fractions (2 Gy per fraction) over a 6-week duration. Concurrent temozolomide (TMZ) chemotherapy was administered orally at a daily dose of 75 mg/m^2^ throughout the radiotherapy course. Adjuvant maintenance TMZ was initiated approximately 4 weeks post-radiotherapy, utilizing a starting dose of 150 mg/m^2^/day for 5 consecutive days within the first 28-day cycle, with subsequent dose escalation to 200 mg/m^2^/day based on individual hematological and non-hematological tolerance. While the baseline adjuvant blueprint encompassed 6 cycles, extension up to 12 cycles was permitted for patients with residual subtotal resections or favorable toxicity profiles.

Radiologically, a solitary tumor was defined as a single contrast-enhancing mass on baseline preoperative MRI scans, whereas multifocal disease was designated by two or more anatomically distinct, non-contiguous contrast-enhancing lesions at presentation. The surgical extent of resection (EOR) was validated via early postoperative MRI performed within 24–48 h. Gross total resection (GTR) was defined by the complete absence of visible residual contrast enhancement, while subtotal resection (STR) was confirmed by persistent postoperative enhancement [25]. Longitudinal treatment response, disease recurrence, and clinical or radiological progression were classified according to the Response Assessment in Neuro-Oncology (RANO) 2.0 guidelines [26]. Corticosteroid use was documented as a binary variable (yes/no) at the time of the initial blood collection. A subset of patients was receiving corticosteroid therapy before frontline treatment. Due to the retrospective cohort of this registry, information on the dose, duration, and dexamethasone-equivalent exposure of corticosteroids was not available.

### 2.4. Determination of Ki-67 Proliferation Index and IDH Status

The Ki-67 proliferation index was quantified via immunohistochemical (IHC) staining performed on formalin-fixed, paraffin-embedded (FFPE) tumor tissue sections using the standard MIB-1 monoclonal antibody (Ventana Medical Systems, Tucson, AZ, USA). The Ki-67 labeling index (LI) was evaluated by senior pathologists using the manual hot-spot method under light microscopy. Briefly, the slides were systematically screened at low magnification to identify areas showing the highest density of nuclear staining (hot spots). Within these representative fields, at least 500 to 1000 tumor cells were counted at high-power magnification (×400), and the Ki-67 index was calculated and expressed as the percentage of positive brownish-stained tumor nuclei relative to the total number of evaluated neoplastic cells.

The molecular status of Isocitrate Dehydrogenase (IDH) was evaluated via immunohistochemical (IHC) staining performed on formalin-fixed, paraffin-embedded (FFPE) tumor tissue sections. Staining was executed using a mutation-specific mouse monoclonal antibody against IDH1 R132H (Clone H09; e.g., Ventana Medical Systems, Tucson, AZ, USA) on an automated slide stainer, according to the manufacturer’s instructions. Cytoplasmic and/or nuclear staining in tumor cells was considered positive for the mutation. Given that the IDH1 R132H alteration accounts for approximately 90% of all IDH mutations in diffuse gliomas, patients demonstrating a definitive absence of immunoreactivity for this specific antibody were classified as IDH-wildtype for the clinical analysis. This surrogate immunohistochemical screening serves as a well-established and highly reliable practical approach in standard neuro-oncological workflows.

### 2.5. Calculation of Inflammatory Indices

Peripheral blood-derived parameters were calculated from a single baseline venous sample collected within 7 to 10 days prior to the initiation of standard chemoradiotherapy. The inflammatory indices were formulated as follows:

- HALP = [Hemoglobin (g/L) × Albumin (g/L) × Lymphocytes (10^9^/L)]/Platelets (10^9^/L)
- H-index = [(Neutrophils × Monocytes)/(Hemoglobin × Albumin × Lymphocytes)] × 100
- AISI = (Neutrophils × Monocytes × Platelets)/Lymphocytes

Notably, the Pan-Immune-Inflammation Value (PIV) and the Aggregate Index of Systemic Inflammation (AISI) utilize identical mathematical formulas derived from absolute peripheral blood counts. However, the term AISI was deliberately selected for this IDH-wildtype Glioblastoma cohort for two primary reasons. First, while the nomenclature “Pan-Immune” implies a universal assessment of all adaptive and innate immune pathways, “AISI” more precisely reflects the cumulative, aggregated burden of systemic inflammatory compartments. Given that IDH-wildtype Glioblastoma pathophysiology is heavily driven by intense, continuous neuro-inflammation and systemic immune suppression, the concept of AISI aligns more accurately with the specific homeostatic disruption under evaluation. Second, the term AISI accentuates the multi-component nature of this index, which integrates major circulating immune lineages to provide a more comprehensive representation of tumor-host interactions compared to traditional single or dual cellular ratios (e.g., NLR or PLR).

### 2.6. Statistical Analyses

Data curation, management, and primary descriptive statistics were performed using IBM SPSS Statistics version 25.0 (IBM Corp., Armonk, NY, USA) and the R software environment version 4.6.1 (R Foundation for Statistical Computing, Vienna, Austria) implemented within the RStudio (Version 2026.08.1) interface utilizing the survival, survminer, rms, and timeROC packages. The normality of continuous data distributions was assessed using the Shapiro–Wilk test. Continuous variables are expressed as medians accompanied by interquartile ranges (IQR), whereas categorical variables are presented as absolute frequencies and corresponding percentages. Intergroup comparisons were evaluated using the Mann–Whitney U test for continuous variables, and the Chi-square (χ^2^) test or Fisher’s exact test for categorical datasets. To establish objective thresholding, maximally selected rank statistics were applied via R to determine the mathematically optimal cut-off values for the HALP score, H-index, and AISI relative to clinical endpoints.

Survival curves were constructed using the non-parametric Kaplan–Meier estimator, and differences between distinct strata were evaluated using the log-rank method. Overall survival (OS) was defined as the temporal interval from the date of primary surgical resection to death from any cause or the date of the last censored follow-up. Progression-free survival (PFS) was calculated from the date of surgery to the documentation of radiological or clinical disease progression, death, or last clinical contact, whichever occurred first. Patients alive and free of progression at the time of final analysis were censored at the date of their last verified clinical assessment.

Variables exhibiting a *p*-value < 0.10 in the univariate screening, alongside clinically established prognostic covariates—including age, biological sex, and the extent of surgical resection (EOR)—were included a priori in multivariate Cox proportional hazards regression models. To prevent multi-collinearity issues stemming from overlapping hematological constituents, each inflammatory index was integrated into the baseline clinical model in separate, independent blocks. To mitigate the risk of type I errors arising from the evaluation of multiple composite scores, a Bonferroni adjustment was applied to the *p*-values of the three index terms, each entered separately into the multivariable Cox models, setting the adjusted significance threshold at α = 0.05/3 = 0.017. Index-specific associations with *p* ≥ 0.017 were interpreted as nominal and not statistically significant. As a supplementary check, the Holm step-down procedure was also applied.

Multi-collinearity diagnostics were monitored using Variance Inflation Factors (VIF), with a threshold of VIF < 5 indicating acceptable non-collinearity. The proportional hazards assumption was rigorously verified through the analysis of Schoenfeld residuals. Internal validation of the final regression models was performed through bootstrapping using 1000 resamples. Because the cut-offs were kept fixed during bootstrapping, this procedure did not account for the optimism associated with cut-off selection. Finally, a series of sensitivity analyses were executed: the inflammation indices were evaluated as log-transformed continuous covariates, age-stratified models were constructed, and time-interaction dynamics were formally tested. Because corticosteroids can directly induce neutrophilia and lymphopenia, an additional sensitivity analysis was restricted to patients who were not receiving corticosteroids at the time of blood sampling. In this subgroup, the cut-offs derived in the full cohort were retained, and the indices were analyzed both as dichotomized and as log-transformed continuous variables. Overall survival models were adjusted for age, sex, extent of resection, comorbidities, and smoking status, and PFS models were additionally adjusted for tumor diameter. Model discrimination and predictive accuracy over time were assessed utilizing inverse probability of censoring weighting (IPCW)-based time-dependent receiver operating characteristic (ROC) curves. All statistical tests were two-tailed, and a *p*-value < 0.05 was considered statistically significant for non-adjusted baseline variables.

## 3. Results

### 3.1. Baseline Clinical and Pathological Characteristics

The retrospective study cohort comprised 136 adult patients with molecularly verified IDH-wildtype Glioblastoma, exhibiting a male predominance and a median age of 57 years. Twenty-five percent of the patients were 65 years of age and older. Concomitant medical comorbidities and a history of active tobacco smoking were prevalent, each documented in over half of the patient population. Mechanistically, maximum safe surgical intervention achieved a high rate of gross total resection (GTR). The primary neoplastic lesions were distributed across the cerebral hemispheres, with the frontal lobe representing the most frequent anatomical site. High proliferative activity, denoted by a Ki-67 index of 30% or higher, and baseline corticosteroid administration prior to frontline chemoradiotherapy were observed in substantial subsets of the cohort. Longitudinal response monitoring according to RANO 2.0 criteria unmasked a high rate of disease recurrence, with a small percentage of relapsed individuals undergoing a secondary surgical procedure. The comprehensive demographics, clinicopathological features, and downstream recurrence statuses are compiled in Table 1.

The cohort was monitored for a mean follow-up duration of 19.1 months (interquartile range [IQR]: 7.7–22.6 months). By the final data lock, 128 patients (94.1%) had died, whereas 8 individuals (5.9%) remained alive. Baseline values of the peripheral blood-derived hematological and biochemical biomarkers are depicted in Table 2.

### 3.2. Prognostic Threshold Optimization via Maximally Selected Rank Statistics

**Figure 2:**
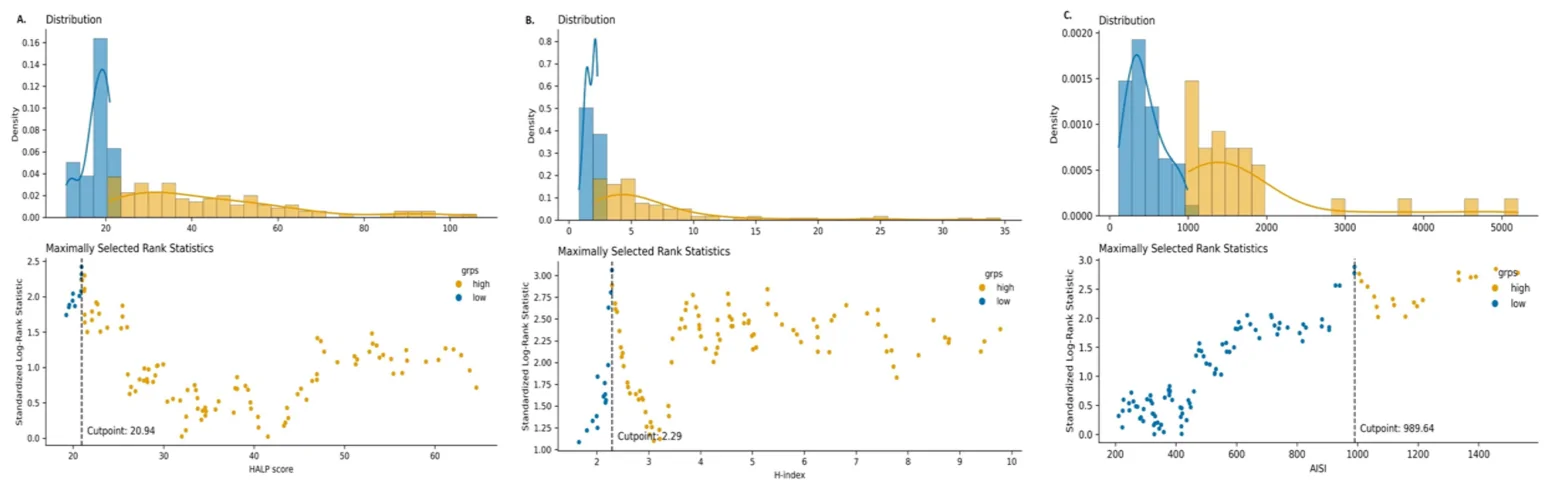
Optimal cut-off value threshold optimization using maximally selected rank statistics. Distribution density histograms (upper panels) and standardized log-rank statistics (lower panels) plotted against continuous baseline values for (**A**) the HALP score, (**B**) the H-index, and (**C**) the aggregate index of systemic inflammation (AISI). Vertical dashed black lines indicate the mathematically optimized prognostic thresholds identified at 20.94, 2.29, and 989.64, respectively.

To mitigate the inherent biases and statistical constraints of conventional dichotomization methods, such as median splits, maximally selected rank statistics were implemented to derive objective, data-driven prognostic cut-offs for each continuous peripheral metric against OS. By leveraging the peak standardized log-rank statistics, this rigorous analytical approach successfully pinpointed optimal mathematical change-points, thereby stratifying the IDH-wildtype Glioblastoma population into discrete high- and low-exposure cohorts (Figure 2).

As illustrated in Figure 2A, the standardized log-rank statistic for the HALP score achieved peak prognostic discrimination at a cut-off value of 20.94. The corresponding distribution density histogram validates that this threshold effectively segregates the cohort into two clinically meaningful strata mirroring nutritional-inflammatory reserves. For the novel H-index (Figure 2B), the optimal prognostic change-point was identified at a threshold of 2.29, representing the most statistically stable peak in the standardized log-rank trajectory. The severe right-skewed non-normal distribution observed in the upper panel highlights that a substantial proportion of patients present with elevated H-index values at baseline, accurately capturing the robust myeloid-biased systemic inflammatory state inherent to IDH-wildtype Glioblastoma progression.

Concurrently, the AISI demonstrated a sharp parametric peak at an optimized threshold of 989.64 (Figure 2C). The clear plateau and subsequent decline in the lower panel confirm that this cutpoint serves as a rigid biological threshold, maximizing the survival discrepancy between patients with low and high pan-inflammatory burdens.

Baseline clinicopathological features were balanced and statistically comparable between the low (<20.94) and high (≥20.94) HALP score cohorts. Conversely, for the H-index and AISI stratifications, a statistically significant discrepancy emerged concerning baseline corticosteroid distribution (*p* < 0.001) (Table 3).

Overall, the individual discriminative capacity of the examined indices was systematically lower for PFS than for OS (Table 4). In univariate OS estimators, all baseline parameters demonstrated a statistically significant association with survival outcomes (HALP: *p* = 0.003; H-index: *p* = 0.007; AISI: *p* < 0.001). For PFS estimation, however, the HALP score was the sole parameter to reach statistical significance (*p* = 0.048).

Longitudinal prognostic accuracy was modeled using inverse probability of censoring weighting (IPCW)-based time-dependent receiver operating characteristic (ROC) curves based on continuous values. The algebraic sign for the HALP score was mathematically inverted to reflect that lower values correspond to increased clinical risk. For PFS estimation, an incremental rise in the area under the curve (AUC) over time was observed specifically for the H-index (AUC values of 0.556 and 0.605 at 6 and 12 months, respectively). For OS estimation, the 6-month and 12-month AUC values remained parallel and stable (Table 5). Crucially, these modest standalone AUC findings indicate that these peripheral indices do not function as isolated predictive modalities; rather, they serve as valuable risk-stratification markers that complement standard clinical variables.

### 3.3. Survival and Covariate Analysis

The median OS for the entire study population was 15.6 months (95% confidence interval [CI]: 14.1–17.3 months), and the median PFS was 9.9 months (95% CI: 8.5–11.4 months). Longitudinal time-to-event outcomes stratified by the mathematically optimized peripheral thresholds were systematically modeled utilizing the non-parametric Kaplan–Meier estimator.

**Figure 3:**
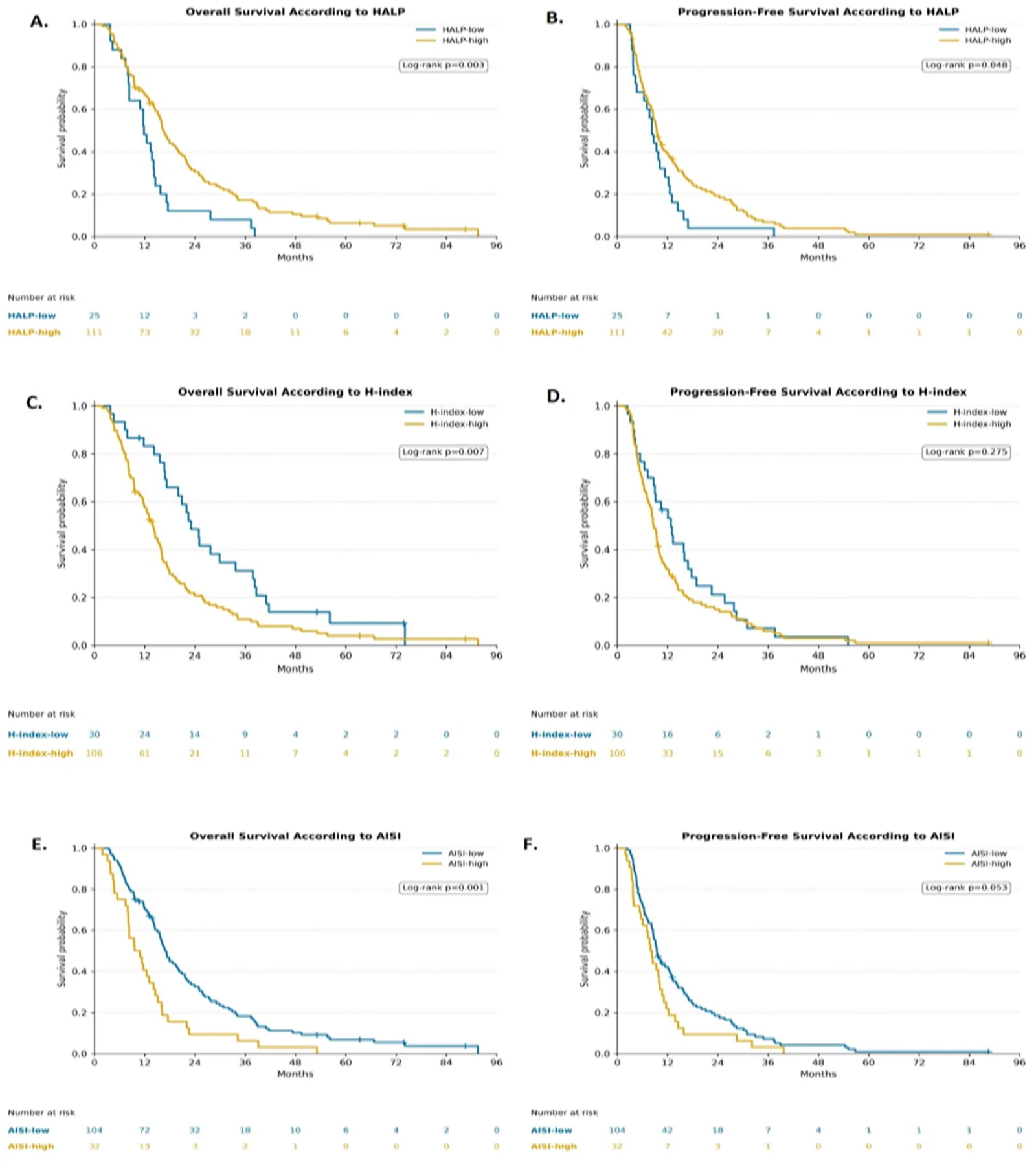
Kaplan–Meier survival trajectories stratified by mathematically optimized baseline bio-index thresholds. The left column displays overall survival (OS) profiles, and the right column illustrates progression-free survival (PFS) frameworks. (**A**) OS and (**B**) PFS trajectories stratified by the hemoglobin-albumin-lymphocyte-platelet (HALP) score threshold (20.94), showing significant separation of both OS and PFS curves in univariate log-rank analysis (**C**) OS and (**D**) PFS curves stratified by the novel H-index threshold (2.29), demonstrating highly significant early mortality discrimination in the overall survival cohort. (**E**) OS and (**F**) PFS trajectories stratified by the aggregate index of systemic inflammation (AISI) threshold (989.64), unmasking a sharp, catastrophic reduction in long-term cumulative survival for patients with elevated pan-inflammatory burdens. Corresponding risk tables (Number at risk) tracking longitudinal follow-up, attrition patterns, and patient censorship are aligned beneath each respective time-to-event panel.

As compiled in the collective survival framework (Figure 3), distinct prognostic divergence was documented between the respective low-exposure and high-exposure cohorts across all evaluated biological indices. For the nutrient-inflammation axis evaluated via the HALP score, a statistically significant survival advantage was demonstrated in the HALP-high cohort (≥20.94). The median OS reached 16.5 months (95% CI: 14.5–18.4) in the HALP-high group compared with 11.9 months (95% CI: 9.9–13.8) in the HALP-low group (log-rank *p* = 0.003; Figure 3A). The HALP score was the only index associated with PFS in univariate analysis, with a median PFS of 10.5 months (95% CI: 8.4–12.6) for the HALP-high stratum versus 9.8 months (95% CI: 7.8–11.8) for the HALP-low stratum (log-rank *p* = 0.048; Figure 3B).

Regarding the host cellular dynamics tracked by the novel H-index, an elevated baseline index (>2.29) was rigidly predictive of compromised long-term survival. The median OS was markedly prolonged in the H-index-low cohort at 23.1 months (95% CI: 19.0–27.1) compared with 13.9 months (95% CI: 12.1–15.8) in the high-exposure tier (log-rank *p* = 0.007; Figure 3C). However, baseline H-index stratification did not demonstrate a standalone statistically significant divergence regarding disease recurrence intervals, with the trajectories converging in the later phases of follow-up (log-rank *p* = 0.275; Figure 3D).

Parallel to these findings, the pan-inflammatory burden calculated via the AISI unmasked a catastrophic reduction in clinical outcomes for the high-exposure group (>989.64). The median OS plummeted from 16.9 months (95% CI: 14.9–18.8) in the AISI-low cohort to a mere 9.6 months (95% CI: 5.4–13.8) in the AISI-high cohort (log-rank *p* < 0.001; Figure 3E). For progression screening, the AISI-high group exhibited a borderline adverse statistical trend toward early recurrence, though it did not cross the conventional significance alpha threshold (log-rank *p* = 0.053; Figure 3F).

In univariate Cox regression analysis, several clinical factors were associated with compromised PFS, including the presence of comorbidities, subtotal surgical resection (failure to achieve GTR), low baseline HALP scores. Correspondingly, poor OS was significantly associated with advanced age, concomitant comorbidities, subtotal resection, baseline corticosteroid use, low HALP scores, elevated H-index, and elevated AISI values (Table 6).

### 3.4. Multivariate Modeling and Validation

Upon multivariable Cox proportional hazards adjustment, the failure to achieve GTR emerged as the sole independent adverse feature for PFS (*p* = 0.004). For overall survival, the presence of comorbidities (*p* = 0.043), subtotal surgical resection (*p* < 0.001), baseline corticosteroid administration (*p* = 0.040), and a low HALP score (*p* = 0.012) were independently associated with poor clinical outcomes. A high AISI value showed a nominal association with OS (*p* = 0.036) that did not meet the Bonferroni-adjusted threshold, and the association for a high H-index did not reach statistical significance (*p* = 0.084) (Table 6). The same conclusions were obtained with the Holm procedure (adjusted *p* = 0.035 for HALP, 0.072 for AISI, and 0.084 for the H-index).

To mitigate potential overfitting and rigorously test stability, a parsimonious model was engineered for each of the three parameters, where hazard ratios correspond to a one-unit increase in the log-transformed continuous index. Parsimonious models were adjusted for age, sex, and extent of resection, while full models additionally controlled for comorbidities, smoking status, and steroid therapy. Continuous significance was maintained for the log-transformed H-index (*p* = 0.013) and AISI (*p* = 0.046) within the parsimonious OS frameworks (Table 7).

Due to a detected violation of the proportional hazards assumption for the age covariate in Schoenfeld residual analysis, multivariable OS estimations were re-evaluated using age-group stratification. These stratified models were adjusted for sex, extent of resection, comorbidities, smoking status, and corticosteroid use. The principal multivariate findings remained stable, and a low HALP score retained its standing as a powerful independent predictor of mortality (*p* = 0.006) (Table 8).

Finally, because the H-index demonstrated a borderline violation of the proportional hazards assumption in Schoenfeld diagnostic testing, a complementary Cox model was constructed by introducing an H-index × log(time) interaction term (Table 9). This framework adjusted for age, sex, extent of resection, comorbidities, smoking, and steroid use. The interaction term did not reach statistical significance (*p* = 0.061). The direction of the coefficients suggested that the hazard associated with an elevated H-index may be higher during the early follow-up period and may decrease over time. However, the main-effect estimate was imprecise (HR: 10.77; 95% CI: 1.25–92.45), and this pattern should therefore be interpreted as hypothesis-generating.

### 3.5. Sensitivity Analysis According to Corticosteroid Exposure

To address the potential confounding effect of corticosteroids, the analyses were repeated in the 80 patients (58.8%) who were not receiving corticosteroids at the time of blood sampling (73 deaths and 76 progression events), using the cut-offs derived in the full cohort (Table 10 and Table 11). In this subgroup, patients with a high H-index had a shorter median OS than those with a low H-index (14.4 vs. 24.9 months, log-rank *p* = 0.025), and the adjusted HR was similar to that observed in the full cohort (HR: 1.60; 95% CI: 0.94–2.72; *p* = 0.082 vs. HR: 1.54; 95% CI: 0.94–2.52; *p* = 0.084). Similarly, patients with a low HALP score had a shorter median OS (12.2 vs. 19.7 months, log-rank *p* = 0.025), and the adjusted HR was nearly unchanged (HR: 1.83; 95% CI: 0.98–3.42; *p* = 0.058), although the confidence interval widened in line with the smaller sample size. In contrast, the association between high AISI and OS was not retained (HR: 0.96; 95% CI: 0.43–2.16; *p* = 0.919). However, only 8 patients in this subgroup had high AISI values, which limits the interpretation of this estimate. None of the indices was associated with PFS in this subgroup. When analyzed as log-transformed continuous variables, none of the indices was significantly associated with OS or PFS, and the continuous association of the H-index observed in the full cohort was attenuated (HR: 1.10; 95% CI: 0.73–1.67; *p* = 0.652).

## 4. Discussion

A substantial body of literature demonstrates that systemic inflammation plays an indispensable role in tumor pathogenesis, actively orchestrating the initiation, progression, angiogenesis, and subsequent metastatic colonization of various malignancies [5,6]. Rather than being a passive byproduct of disease progression, the host systemic inflammatory response, coupled with nutritional homeostasis, forms a complex interconnected axis that heavily modulates survival outcomes [7,8,27]. In neuro-oncology, this relationship is particularly vital due to the unique cellular architecture of the central nervous system, where glioblastoma cells induce an immunosuppressive, myeloid-biased microenvironment [9,15].

To the best of our knowledge, the present study represents the pioneer clinical investigation to evaluate the prognostic significance of the H-index in individuals diagnosed with IDH-wildtype Glioblastoma, while concurrently validating the clinical utility of the HALP score and the AISI. The principal finding of this retrospective registry is that peripheral blood-derived indices provide prognostic information in IDH-wildtype Glioblastoma. A low baseline H-index was associated with prolonged OS in univariate analysis, and this separation persisted among patients not receiving corticosteroids. A low HALP score was independently associated with poor OS. The H-index did not reach statistical significance in multivariable analysis, and the association for AISI did not remain significant after correction for multiple testing. These data strongly reinforce the growing oncology paradigm that host-specific systemic inflammatory cascades and nutritional reserves heavily modulate the clinical trajectory of high-grade gliomas.

Crucially, the prognostic value of the H-index and AISI in our cohort transcends a mere reflection of general physical frailty or cachexia, deeply aligning instead with the distinct immunobiology of the IDH-wildtype Glioblastoma tumor microenvironment (TME). This tumor is notoriously characterized as an immunologically ‘cold’ tumor, dominated not by adaptive T-cell infiltrates but rather by a profound accumulation of bone marrow-derived myeloid cells, which can constitute up to 40% of the total tumor mass. The absolute neutrophil and monocyte counts that drive the numerators of both the H-index and AISI directly mirror this systemic myeloid-biased polarization. Preclinical and translational evidence indicates that IDH-wildtype Glioblastoma cells continuously secrete chemokine ligands such as CCL2, CSF-1, and transforming growth factor-beta (TGF-β), which actively recruit circulating monocytes and neutrophils, converting them intratumorally into immunosuppressive tumor-associated macrophages (TAMs) and myeloid-derived suppressor cells (MDSCs), as detailed by Virtuoso et al. [5]. These myeloid subsets directly promote vascular endothelial growth factor (VEGF)-mediated angiogenesis, degrade the extracellular matrix, and induce profound T-cell exhaustion, thereby fueling aggressive tumor kinetics and treatment resistance, a phenomenon strongly supported by Hanggi et al. [9]. Consequently, rather than functioning as generic surrogates of systemic illness, an elevated baseline H-index or AISI specifically indexes the intensity of the host’s systemic myeloid drive that sustains the IDH-wildtype Glioblastoma’s immunosuppressive niche. While our retrospective data demonstrate robust clinical associations rather than direct biological causation, these indices offer a reliable peripheral window into the intra-tumoral immunosuppressive cross-talk.

During later clinical phases, local factors within the tumor microenvironment—such as intense tissue hypoxia, radiation-induced necrosis, disrupted blood–brain barrier permeability, and hypermutated clonal evolution—likely overshadow the systemic baseline inflammatory signals captured at initial blood analyses. This biological shift indicates that host peripheral systemic immunotolerance is highly informative during early treatment stages but yields to local spatial heterogeneity and intrinsic tumor mechanics over time [4,5].

An additional exploratory observation concerns the time-dependent behavior of the H-index. In the model including an H-index × log(time) interaction term, the hazard associated with a high H-index appeared to be higher during the early follow-up period and to decrease over time. However, the interaction term did not reach statistical significance (HR: 0.51, *p* = 0.061), and the main-effect estimate was imprecise (HR: 10.77; 95% CI: 1.25–92.45). This pattern should therefore be interpreted as hypothesis-generating. The H-index was originally defined by Valero et al. in oral cavity carcinomas [22] and later validated by Boscolo-Rizzo et al. in surgically treated laryngeal squamous cell carcinoma [28], where an elevated score rigidly predicted poor 5-year survival. More recently, a multicenter cohort study by Seyyar et al. extended its utility to early-stage HER2-positive breast cancer, demonstrating that a low baseline H-index (using a nearly identical cut-off of 2.3) independently predicts a superior pathological complete response [23]. Kattepur et al. have similarly highlighted its emerging role as a powerful prognostic marker in gallbladder cancer [24].

Patients with elevated baseline AISI values had a shorter median OS in univariate analysis (*p* < 0.001). However, the multivariable association (HR: 1.65, *p* = 0.036) did not meet the Bonferroni-adjusted threshold and was not retained among patients not receiving corticosteroids. Because 75.0% of patients with high AISI values were receiving corticosteroids, the prognostic signal of AISI in our cohort may be partly attributable to corticosteroid exposure. While specific AISI literature in IDH-wildtype Glioblastoma remains sparse, our findings align robustly with recent large-scale meta-analyses investigating similar pan-inflammatory scores in gliomas. For instance, comprehensive analyses by Zhang and Ni confirmed that elevated Systemic Immune-Inflammation Index (SII) and Systemic Inflammation Response Index (SIRI) scores universally predict dismal OS and PFS in glioma cohorts, demonstrating a significant correlation with high Ki-67 proliferation indexes [15].

Biologically, AISI aggregates neutrophils, monocytes, and platelets in its numerator, which are the main cellular components driving tumor-associated immunosuppression. Elevated circulating monocytes and neutrophils promote the secretion of immunosuppressive cytokines and matrix metalloproteinases, transforming the immunologically “cold” IDH-wildtype Glioblastoma microenvironment into a permissive niche for pathological neovascularization and local tissue invasion. Crucially, as recently shown by Şahin et al. in a longitudinal analysis, inflammatory biomarkers remain consistently higher in patients who undergo subtotal resection (STR) compared to gross total resection (GTR), reflecting the presence of residual tumor tissue and a persistent inflammatory burden throughout the treatment course [25]. This matches our observation that composite indices derived from multiple circulating immune lineages provide a far superior representation of tumor-host interactions than traditional single or dual cellular ratios.

In contrast to the H-index and AISI, which are purely inflammation-driven, the HALP score incorporates hemoglobin and albumin, thus bridging systemic inflammation with the host’s nutritional reserves and metabolic performance. In our cohort, the HALP score was the only index associated with PFS in univariate analysis (*p* = 0.048), although this association was not retained in the multivariable model (*p* = 0.172). The HALP score was the only index that remained independently associated with OS after correction for multiple testing (HR: 1.82, *p* = 0.012). This outcome is directly congruent with a recent landmark study by Demir et al., which evaluated the HALP score as a predictor of survival in IDH-wildtype Glioblastoma and established its prognostic value in identifying high-risk patients [19]. Furthermore, our findings replicate data from Korkmaz et al. in recurrent IDH-wildtype Glioblastoma cohorts treated with second-line regimens (such as bevacizumab plus irinotecan), where baseline HALP scores stood out as powerful independent predictors of both PFS and OS [29].

Hypoalbuminemia and anemia are documented surrogates for systemic wasting, chronic malnutrition, and tumor-mediated cachexia. Low serum albumin concentrations mirror an ongoing acute-phase response driven by sustained tumor-derived inflammation, whereas anemia compromises tissue oxygenation, worsening the intra-tumoral hypoxic stress that triggers glioblastoma stem cell activation and treatment resistance. Recently, Balci Topuz and Ozturk Iyilikci demonstrated that other multi-component nutritional-inflammatory scores, such as the CALLY index, provide improved prognostic stratification compared with other cell-count scores in IDH-wildtype Glioblastoma treated with the Stupp protocol [20]. This collective evidence supports our finding that patients who preserve robust nutritional baselines tolerate standard concurrent chemoradiotherapy more favorably, thereby avoiding early treatment interruptions and achieving delayed disease progression.

Because corticosteroids increase circulating neutrophils and decrease lymphocytes, and both cell types are components of the H-index and AISI, corticosteroid exposure is an important potential confounder in studies evaluating these indices. In our cohort, corticosteroid use was unevenly distributed between the H-index and AISI groups. In the sensitivity analysis restricted to patients not receiving corticosteroids, the survival separation according to the H-index cut-off and the magnitude of the adjusted hazard ratio were largely preserved, whereas the continuous association was attenuated and the AISI association was not retained. These findings suggest that the dichotomized H-index may carry prognostic information that is not fully explained by corticosteroid exposure, but they do not allow residual confounding to be excluded. The HALP score, whose groups were balanced for corticosteroid use, showed the most consistent association with OS across the analyses.

Despite the clinical relevance of our findings, certain limitations must be addressed, primarily stemming from the retrospective, single-center architecture of this registry. First, corticosteroid exposure is an important confounding factor. Glucocorticoids are well known to induce neutrophilia via bone marrow demargination and to provoke lymphopenia through accelerated apoptosis, potentially inflating baseline inflammation indices. Corticosteroid use was recorded only as a binary variable at the time of blood sampling, and information on dose, duration, and dexamethasone-equivalent exposure was not available. Therefore, dose-based analyses could not be performed. Although a sensitivity analysis restricted to patients not receiving corticosteroids was performed, this subgroup was relatively small, particularly for the high-AISI group (*n* = 8), which limits statistical power, and residual confounding cannot be excluded. Second, the cut-offs were derived and evaluated within the same dataset using maximally selected rank statistics. Bootstrap internal validation was performed with fixed cut-offs and did not account for the optimism associated with cut-off selection. Therefore, the discrimination estimates and the stability of the thresholds may be overestimated, and in the absence of an independent validation cohort, these cut-offs should be regarded as exploratory and cohort-specific. Third, certain inherent constraints of this retrospective registry must be addressed. Granular clinical documentation regarding the exact numerical distribution of Karnofsky Performance Status (KPS) scores was not uniformly retrievable from historical medical records. While all included patients strictly met the functional baseline threshold of KPS ≥ 70—ensuring a homogeneous cohort capable of completing intensive frontline Stupp protocols—individualized performance modeling was unfeasible. Similarly, key molecular variables, most notably MGMT promoter methylation status, were verified in exactly 10 out of the 136 patients (7.3%) due to shifting diagnostic infrastructures and institutional protocols between 2014 and 2025. From a methodological standpoint, performing multivariable adjustments or subgroup analyses on a parameter with such extensive missingness (>92%) would induce catastrophic selection bias, cause extreme model overfitting, and fundamentally compromise the statistical power of our primary outcomes and 1000-resample bootstrapping. Consequently, these parameters were deliberately omitted to preserve the mathematical integrity of the observed peripheral biomarker signals, presenting a clear avenue for future prospective biobanking trials.

The consistency of the HALP findings across bootstrap internal validation, age-stratified models, and the corticosteroid-restricted sensitivity analysis supports the internal consistency of this result. Future multicenter, prospective studies with structured documentation of corticosteroid dose and timing, as well as longitudinal blood sampling, are needed to externally validate these cut-offs and to clarify the underlying biological mechanisms.

## 5. Conclusions

The H-index, evaluated here for the first time in IDH-wildtype Glioblastoma, was associated with OS in univariate analysis and among patients not receiving corticosteroids, and the HALP score was independently associated with OS. However, the H-index did not reach independent statistical significance, and the cut-offs identified in this study are exploratory and cohort-specific. These readily available peripheral indices may complement conventional clinical prognostic factors, but their clinical utility should be confirmed in independent prospective cohorts before they are considered for routine clinical practice.

## Figures and Tables

**Table 1:** Baseline Demographics and Clinicopathological Characteristics of the IDH-wildtype Glioblastoma Cohort (*N* = 136).

Variable	Category	No. (%)	Value/Range
Age (years)	Mean ± SD		57.0 ± 13.4
	Range		18.0–80.0
Sex	Male	84 (61.8)	
	Female	52 (38.2)	
Comorbidities	Present	73 (53.7)	
	Absent	63 (46.3)	
Smoking Status	Yes	74 (54.4)	
	No	62 (45.6)	
Extent of Resection (EOR)	GTR	101 (74.3)	
	STR	35 (25.7)	
Tumor Location	Frontal	49 (36.0)	
	Temporal	38 (27.9)	
	Parietal	36 (26.5)	
	Occipital	13 (9.6)	
Cerebral Hemisphere	Right	68 (50.0)	
	Left	68 (50.0)	
Multifocality	Present	8 (5.9)	
	Absent	128 (94.1)	
Tumor Diameter (mm)	Mean ± SD		50.0 ± 13.8
	Range		10.0–90.0
Ki-67 Index (%)	Mean ± SD		30.0 ± 18.3
	Range		10.0–90.0
Corticosteroid Use	Yes	56 (41.2)	
	No	80 (58.8)	
Anticonvulsant Use	Yes	128 (94.1)	
	No	8 (5.9)	
Recurrence Status	Present	118 (86.8)	
	Absent	18 (13.2)	
Repeat Surgery at Progression	Yes	18 (13.2)	
	No	118 (86.8)	

Note: Data are presented as absolute numbers with percentages in parentheses, or as mean values with standard deviations, where appropriate. Abbreviations: GTR, gross total resection; *N*, total number of patients; SD, standard deviation; STR, subtotal resection.

**Table 2:** Baseline Clinical Values of Peripheral Blood-Derived Inflammatory Biomarkers.

Biomarker	Mean ± SD	Median (Range)	IQR
HALP	39.2 ± 20.9	33.8 (10.8–106.1)	22.2–51.2
H-index	5.6 ± 5.6	4.0 (0.8–34.6)	2.4–6.4
AISI	758.2 ± 757.3	519.6 (115.0–5203.5)	323.5–977.3

Note: Data are presented as mean values with standard deviations, or as medians with ranges/interquartile ranges, where appropriate. Abbreviations: AISI, aggregate index of systemic inflammation; HALP, hemoglobin, albumin, lymphocyte, and platelet; IQR, interquartile range; SD, standard deviation.

**Table 3:** Patient Stratification and Comparative Baseline Analysis Across HALP, H-index, and AISI Thresholds.

Variable	Category/Statistic	HALP			H-Index			AISI		
		Low (≤20.94) (*n* = 25)	High (>20.94) (*n* = 111)	*p*-Value	Low (≤2.29) (*n* = 30)	High (>2.29) (*n* = 106)	*p*-Value	Low (≤989.64) (*n* = 104)	High (>989.64) (*n* = 32)	*p*-Value
Age (years)	Mean ± SD Range	56.6 ± 11.9 26–76	53.6 ± 13.7 18–80	0.276	51.9 ± 14.8 22–76	54.7 ± 13.0 18–80	0.350	53.8 ± 14.1 18–80	55.2 ± 10.7 26–72	0.555
Sex	Male Female	12 (48.0) 13 (52.0)	72 (64.9) 39 (35.1)	0.180	15 (50.0) 15 (50.0)	69 (65.1) 37 (34.9)	0.197	60 (57.7) 44 (42.3)	24 (75.0) 8 (25.0)	0.120
Comorbidities	Yes No	15 (60.0) 10 (40.0)	58 (52.3) 53 (47.7)	0.631	14 (46.7) 16 (53.3)	59 (55.7) 47 (44.3)	0.506	51 (49.0) 53 (51.0)	22 (68.8) 10 (31.3)	0.080
Smoking Status	Yes No	12 (48.0) 13 (52.0)	62 (55.9) 49 (44.1)	0.624	12 (40.0) 18 (60.0)	62 (58.5) 44 (41.5)	0.112	53 (51.0) 51 (49.0)	21 (65.6) 11 (34.4)	0.210
EOR	GTR STR	17 (68.0) 8 (32.0)	84 (75.7) 27 (24.3)	0.589	24 (80.0) 6 (20.0)	77 (72.6) 29 (27.4)	0.564	80 (76.9) 24 (23.1)	21 (65.6) 11 (34.4)	0.295
Anatomical Location	Frontal Temporal Parietal Occipital	12 (48.0) 6 (24.0) 5 (20.0) 2 (8.0)	37 (33.3) 32 (28.8) 31 (27.9) 11 (9.9)	0.624	8 (26.7) 8 (26.7) 11 (36.7) 3 (10.0)	41 (38.7) 30 (28.3) 25 (23.6) 10 (9.4)	0.473	37 (35.6) 29 (27.9) 28 (26.9) 10 (9.6)	12 (37.5) 9 (28.1) 8 (25.0) 3 (9.4)	1.000
Tumor Hemisphere	Right Left	11 (44.0) 14 (56.0)	57 (51.4) 54 (48.6)	0.658	16 (53.3) 14 (46.7)	52 (49.1) 54 (50.9)	0.836	55 (52.9) 49 (47.1)	13 (40.6) 19 (59.4)	0.312
Multifocality	Yes No	1 (4.0) 24 (96.0)	7 (6.3) 104 (93.7)	1.000	2 (6.7) 28 (93.3)	6 (5.7) 100 (94.3)	1.000	7 (6.7) 97 (93.3)	1 (3.1) 31 (96.9)	0.680
Tumor Diameter (mm)	Mean± SD Range	52.8 ± 12.2 30–78	48.8 ± 14.1 10–90	0.154	50.5 ± 13.1 23–73	49.3 ± 14.0 10–90	0.656	48.4 ± 13.6 10–77	53.2 ± 14.0 30–90	0.093
Ki-67 Index (%)	Mean± SD Range	36.4 ± 16.5 15–80	35.4 ± 18.7 10–90	0.783	35.3 ± 21.4 15–90	35.6 ± 17.4 10–90	0.948	35.5 ± 18.3 10–90	35.8 ± 18.4 10–80	0.936
Cortico- steroid Use	Yes No	11 (44.0) 14 (56.0)	45 (40.5) 66 (59.5)	0.926	1 (3.3) 29 (96.7)	55 (51.9) 51 (48.1)	**<0.001**	32 (30.8) 72 (69.2)	24 (75.0) 8 (25.0)	**<0.001**
Anticonvulsants Use	Yes No	25 (100.0) 0 (0.0)	103 (92.8) 8 (7.2)	0.351	26 (86.7) 4 (13.3)	102 (96.2) 4 (3.8)	0.071	97 (93.3) 7 (6.7)	31 (96.9) 1 (3.1)	0.680
Secondary Surgery	Yes No	1 (4.0) 24 (96.0)	17 (15.3) 94 (84.7)	0.194	7 (23.3) 23 (76.7)	11 (10.4) 95 (89.6)	0.074	14 (13.5) 90 (86.5)	4 (12.5) 28 (87.5)	1.000
Recurrence Status	Present Absent	20 (80.0) 5 (20.0)	98 (88.3) 13 (11.7)	0.325	27 (90.0) 3 (10.0)	91 (85.8) 15 (14.2)	0.763	90 (86.5) 14 (13.5)	28 (87.5) 4 (12.5)	1.000
Median OS (months,95% CI)		11.9 (9.9–13.8)	16.5 (14.5–18.4)	**0.003**	23.1 (19.0–27.1)	13.9 (12.1–15.8)	**0.007**	16.9 (14.9–18.8)	9.6 (5.4–13.8)	**<0.001**
Median PFS (months,95% CI)		9.8 (7.8–11.8)	10.5 (8.4–12.6)	**0.048**	13.3 (8.7–17.9)	9.6 (8.3–10.9)	0.275	10.7 (7.8–13.5)	9.3 (6.3–12.4)	**0.053**

Notes: Categorical variables are reported as absolute numbers with percentages in parentheses, *n* (%). Continuous distributions are presented as mean ± standard deviation (SD) along with full ranges. Intergroup differences were assessed using Mann–Whitney U tests for continuous variables, and Chi-square (χ^2^) or Fisher’s exact tests for categorical data as appropriate. Statistically significant *p*-values (*p* < 0.05) are highlighted in bold. Abbreviations: AISI: Aggregate index of systemic inflammation; CI: Confidence Interval; EOR: Extent of Resection; GTR: Gross Total Resection; HALP: Hemoglobin, Albumin, Lymphocyte, and Platelet score; OS: Overall Survival; PFS: Progression-Free Survival; STR: Subtotal Resection.

**Table 4:** Log-Rank Significance Analysis for PFS and OS Based on Mathematically Optimized Thresholds.

Biomarker	Cut-Off Value	Cohort Distribution, n (%) (Low/High)	PFS Estimation (Log-Rank *p*-Value)	OS Estimation (Log-Rank *p*-Value)
HALP	20.94	25 (18.3)/111 (81.7)	**0.048**	**0.003**
H-index	2.29	30 (22.0)/106 (78.0)	0.275	**0.007**
AISI	989.64	104 (76.5)/32 (23.5)	0.053	**<0.001**

Note: Statistically significant values (*p* < 0.05) are highlighted in bold. Abbreviations: AISI: Aggregate index of systemic inflammation; HALP: Hemoglobin, Albumin, Lymphocyte, and Platelet score; OS: Overall Survival; PFS: Progression-Free Survival.

**Table 5:** Inverse Probability of Censoring Weighting (IPCW)-Adjusted Time-Dependent ROC Analysis for OS and PFS Outcomes.

Biomarker	Overall Survival (OS)		Progression-Free Survival (PFS)	
	AUC at 6 Months (*95% CI*)	AUC at 12 Months (*95% CI*)	AUC at 6 Months (*95% CI*)	AUC at 12 Months (*95% CI*)
HALP	0.596 (0.462–0.731)	0.551 (0.447–0.654)	0.545 (0.438–0.651)	0.513 (0.414–0.612)
H-index	0.652 (0.495–0.808)	0.628 (0.530–0.725)	0.556 (0.449–0.662)	0.605 (0.507–0.703)
AISI	0.635 (0.478–0.793)	0.625 (0.526–0.724)	0.535 (0.429–0.642)	0.557 (0.459–0.656)

Note: Data represent time-dependent Area Under the Curve (AUC) statistics computed via inverse probability of censoring weighting (IPCW) to account for longitudinal clinical censoring. Abbreviations: AISI: Aggregate index of systemic inflammation; AUC: Area Under the Curve; CI: Confidence Interval; HALP: Hemoglobin, Albumin, Lymphocyte, and Platelet score. Note: The mathematical sign for the HALP score was inverted during analytical modeling because depressed levels of this index denote increased clinical risk.

**Table 6:** Univariate and Multivariate Cox Proportional Hazards Regression Analyses for PFS and OS Outcomes.

Variable	PFS			OS		
	Univariate *p*-Value	Multivariate *p*-Value	Hazard Ratio (*95% CI*)	Univariate *p*-Value	Multivariate *p*-Value	Hazard Ratio (*95% CI*)
Age (years) (≥*57* vs. <*57 [Ref.]*)	0.053	0.260	1.27 (0.84–1.91)	**0.026**	0.454	1.17 (0.78–1.77)
Biological Sex (*Male* vs. *Female [Ref.]*)	0.587	0.203	0.61 (0.29–1.30)	0.344	0.839	0.92 (0.43–1.98)
Comorbidities (*Yes* vs. *No [Ref.]*)	**0.048**	0.607	1.12 (0.73–1.71)	**0.005**	**0.043**	1.54 (1.01–2.33)
Smoking Status (*Yes* vs. *No [Ref.]*)	0.100	0.061	2.04 (0.97–4.28)	0.084	0.453	1.33 (0.63–2.83)
Extent of Resection (EOR) (*STR* vs. *GTR [Ref.]*)	**<0.001**	**0.004**	1.88 (1.22–2.88)	**<0.001**	**<0.001**	2.07 (1.37–3.13)
Tumor Location (*Non-frontal* vs. *Frontal [Ref.]*)	0.325	—	—	0.292	—	—
Cerebral Hemisphere (*Left* vs. *Right [Ref.]*)	0.998	—	—	0.796	—	—
Multifocality (*Yes* vs. *No [Ref.]*)	0.608	—	—	0.348	—	—
Tumor Diameter (mm) (*Continuous*)	0.056	0.219	1.01 (1.00–1.02)	0.330	—	—
Ki-67 Proliferation Index (%) (*Continuous*)	0.264	—	—	0.974	—	—
Baseline Corticosteroid Use (*Yes* vs. *No [Ref.]*)	0.103	—	—	**0.037**	**0.040**	1.47 (1.02–2.12)
Anticonvulsant Therapy (*Yes* vs. *No [Ref.]*)	0.597	—	—	0.964	—	—
HALP Score (*Low* vs. *High [Ref.]*)	**0.048**	0.172	1.37 (0.87–2.15)	**0.003**	**0.012**	1.82 (1.14–2.89)
H-index (*High* vs. *Low [Ref.]*)	0.275	—	—	**0.007**	0.084	1.54 (0.94–2.52)
AISI (*High* vs. *Low [Ref.]*)	0.053	0.357	1.23 (0.79–1.92)	**<0.001**	0.036	1.65 (1.03–2.64)

Note: Statistically significant values are highlighted in bold (*p* < 0.05 for clinical covariates, and *p* < 0.017 after Bonferroni correction for the three inflammatory indices). Holm-adjusted *p*-values for the indices in the OS models were 0.035 (HALP), 0.072 (AISI), and 0.084 (H-index). Variables with *p* < 0.10 in univariate models were integrated a priori into multivariable models. Due to shared hematological constituents, each inflammatory index was entered into the multivariable baseline clinical model in independent, separate analytical blocks. Abbreviations: AISI: Aggregate index of systemic inflammation; CI: Confidence Interval; GTR: Gross Total Resection; HALP: Hemoglobin, Albumin, Lymphocyte, and Platelet score; OS: Overall Survival; PFS: Progression-Free Survival; Ref.: Reference category; STR: Subtotal Resection.

**Table 7:** Cox Proportional Hazards Regression Sensitivity Analyses Utilizing Log-Transformed Continuous Indices.

Log-Transformed Biomarker	Parsimonious Model: OS		Parsimonious Model: PFS		Full Model: OS	
	Hazard Ratio (*95% CI*)	*p*-Value	Hazard Ratio (*95% CI*)	*p*-Value	Hazard Ratio (*95% CI*)	*p*-Value
log(HALP)	0.87 (0.60–1.26)	0.451	0.83 (0.58–1.20)	0.323	0.86 (0.59–1.24)	0.414
log(H-index)	1.43 (1.08–1.90)	**0.013**	1.17 (0.90–1.53)	0.248	1.23 (0.90–1.69)	0.192
log(AISI)	1.29 (1.00–1.65)	**0.046**	1.05 (0.83–1.33)	0.693	1.12 (0.85–1.48)	0.419

Note: Statistically significant values (*p* < 0.05) are highlighted in bold. Hazard ratio (HR) values correspond to a one-unit increase in the natural log-transformed index. Parsimonious Models: Adjusted for baseline clinical covariates including age, sex, and extent of resection (EOR). Full Models: Additionally controlled for age, sex, extent of resection, clinical comorbidities, smoking status, and corticosteroid use. Statistical Note: Because the baseline inflammatory indices exhibited a severe right-skewed non-normal distribution (skewness coefficients: HALP = 1.18; H-index = 2.94; AISI = 3.17; Shapiro–Wilk *p* < 0.001 for all), parametric modeling was strictly performed utilizing natural logarithm transformations to ensure mathematical stability. Abbreviations: AISI: Aggregate index of systemic inflammation; CI: Confidence Interval; HALP: Hemoglobin, Albumin, Lymphocyte, and Platelet score; OS: Overall Survival; PFS: Progression-Free Survival.

**Table 8:** Sensitivity Analysis for Overall Survival Utilizing Age-Stratified Multivariable Cox Proportional Hazards Models.

	Full Model (Unstratified)		Age-Stratified Model	
	Hazard Ratio (*95% CI*)	*p*-Value	Hazard Ratio (*95% CI*)	*p*-Value
HALP (*Low* vs. *High [Ref.]*)	1.82 (1.14–2.89)	**0.012**	1.95 (1.22–3.13)	**0.006**
H-index (*High* vs. *Low [Ref.]*)	1.54 (0.94–2.52)	0.084	1.46 (0.89–2.40)	0.131
AISI (*High* vs. *Low [Ref.]*)	1.65 (1.03–2.64)	**0.036**	1.52 (0.94–2.44)	0.086

Note: Statistically significant values (*p* < 0.05) are highlighted in bold. Model Adjustments: Both the unstratified Full Model and the Age-Stratified Model were adjusted for biological sex, extent of resection (EOR), presence of clinical comorbidities, tobacco smoking status, and baseline corticosteroid use. Statistical Rationale: Due to the violation of the proportional hazards assumption regarding the continuous age variable observed in the primary Schoenfeld residual diagnostic checks, multivariable overall survival frameworks were re-evaluated and validated using direct stratification by age groups. Abbreviations: AISI: Aggregate index of systemic inflammation; CI: Confidence Interval; HALP: Hemoglobin, Albumin, Lymphocyte, and Platelet score; Ref.: Reference category.

**Table 9:** Time-Dependent Effect Analysis via Cox Proportional Hazards Model with Time-Interaction for the H-index Concerning Overall Survival.

Model Component	Hazard Ratio (HR)	*p*-Value
H-index (*High* vs. *Low [Ref.]*) (Main Effect)	10.77	**0.030**
H-index (High) × log(time) (Interaction Term)	0.51	0.061

Note: Statistically significant values (*p* < 0.05) are highlighted in bold. Model Adjustments: The extended Cox proportional hazards framework was adjusted for clinical baseline variables including age, sex, extent of resection (EOR), presence of comorbidities, tobacco smoking status, and baseline corticosteroid administration. The main-effect hazard ratio (95% CI: 1.25–92.45) refers to log(time) = 0, that is, the first month of follow-up. Statistical and Clinical Rationale: Due to a borderline violation of the proportional hazards assumption detected for the H-index covariate in primary Schoenfeld diagnostic checks, a time-interaction term [H-index × log(time)] was introduced. Although the interaction term trended toward significance without crossing the strict alpha threshold (*p* = 0.061), the mathematical direction of the coefficient indicates that the mortality risk linked to an elevated H-index is highly pronounced during the initial follow-up phase and gradually attenuates during long-term clinical monitoring. Abbreviations: HR: Hazard Ratio; Ref.: Reference category.

**Table 10:** Sensitivity Analysis Restricted to Patients Not Receiving Corticosteroids at Blood Sampling: Dichotomized Indices.

Index	Steroid-Free Subgroup (*n* = 80) Kaplan–Meier				Full Cohort (*n* = 136) OS		Steroid-Free OS		Steroid-Free PFS	
	*n* (Low/High)	Median OS, Low (*95% CI*)	Median OS, High (*95% CI*)	Log-Rank *p*-Value	HR (*95% CI*)	*p*-Value	HR (*95% CI*)	*p*-Value	HR (*95% CI*)	*p*-Value
HALP (*Low* vs. *High [Ref.]*)	14/66	12.2 (10.9–37.4)	19.7 (15.9–25.7)	0.025	1.82 (1.14–2.89)	0.012	1.83 (0.98–3.42)	0.058	1.53 (0.82–2.84)	0.183
H-index (*High* vs. *Low [Ref.]*)	29/51	24.9 (20.0–38.3)	14.4 (11.9–18.7)	0.025	1.54 (0.94–2.52)	0.084	1.60 (0.94–2.72)	0.082	1.03 (0.59–1.79)	0.922
AISI (*High* vs. *Low [Ref.]*)	72/8	17.9 (14.5–25.0)	13.3 (10.9–NR)	0.176	1.65 (1.03–2.64)	0.036	0.96 (0.43–2.16)	0.919	0.79 (0.35–1.79)	0.567

Note: Groups were defined using the cut-offs derived in the full cohort (HALP 20.94, H-index 2.29, AISI 989.64). The steroid-free subgroup included 73 deaths and 76 progression events. Full-cohort models were adjusted for age, sex, extent of resection, comorbidities, smoking status, and corticosteroid use. Steroid-free OS models were adjusted for age, sex, extent of resection, comorbidities, and smoking status. Steroid-free PFS models were additionally adjusted for tumor diameter. For the indices, the Bonferroni-adjusted significance threshold was *p* < 0.017. Abbreviations: AISI: aggregate index of systemic inflammation; CI: confidence interval; HALP: hemoglobin, albumin, lymphocyte, and platelet score; HR: Hazard ratio; NR: not reached; OS: overall survival; PFS: progression-free survival; Ref.: reference category.

**Table 11:** Sensitivity Analysis Restricted to Patients Not Receiving Corticosteroids at Blood Sampling: Log-Transformed Continuous Indices.

Log-Transformed Biomarker	Parsimonious Model OS		Parsimonious Model PFS		Full Model OS	
	HR (*95% CI*)	*p*-Value	HR (*95% CI*)	*p*-Value	HR (*95% CI*)	*p*-Value
log (HALP)	0.98 (0.57–1.66)	0.935	0.98 (0.56–1.70)	0.937	1.04 (0.60–1.80)	0.879
log (H-index)	1.10 (0.73–1.67)	0.652	0.84 (0.55–1.27)	0.402	1.02 (0.67–1.55)	0.937
log (AISI)	1.00 (0.71–1.42)	0.979	0.79 (0.56–1.12)	0.185	0.95 (0.67–1.34)	0.777

Note: *n* = 80. Parsimonious models were adjusted for age, sex, and extent of resection. Full models were additionally adjusted for comorbidities and smoking status. Hazard ratios correspond to a one-unit increase in the natural log-transformed index. Abbreviations: AISI: aggregate index of systemic inflammation; CI: confidence interval; HALP: hemoglobin, albumin, lymphocyte, and platelet score; HR: Hazard ratio; OS: overall survival; PFS: progression-free survival.

## Data Availability

The datasets used in this study can be made available by the corresponding author upon reasonable request, with permission from the Medical Oncology Department of Gaziantep University.

## Abbreviations

The following abbreviations are used in this manuscript:

AISI	Aggregate Index of Systemic Inflammation
AUC	Area Under the Curve
CALLY	CRP–Albumin–Lymphocyte Index
CI	Confidence Interval
EOR	Extent of Surgical Resection
GTR	Gross Total Resection
HALP	Hemoglobin–Albumin–Lymphocyte–Platelet
HR	Hazard Ratio
IDH	Isocitrate Dehydrogenase
IPCW	Inverse Probability of Censoring Weighting
NLR	Neutrophil-to-Lymphocyte Ratio
OS	Overall Survival
PFS	Progression-Free Survival
PIV	Pan-immune-inflammation Value
PLR	Platelet-to-Lymphocyte Ratio
ROC	Receiver Operating Characteristic
SII	Systemic Immune-Inflammation Index
SIRI	Systemic Inflammation Response Index
STR	Subtotal Resection
VIF	Variance Inflation Factor
